# 2024–2025 Routine review of SMFM documents

**DOI:** 10.1002/pmf2.70330

**Published:** 2026-06-05

**The following SMFM documents have been withdrawn effective 2024**:

Consult Series #40: The role of routine cervical length screening in selected high‐and low‐risk women for preterm birth prevention

Statement #7: The role of cervical pessary placement to prevent preterm birth in clinical practice

**The following SMFM documents have been withdrawn effective 2025**:

Special Statement: Who's who in patient safety and quality for maternal healthcare in the United States

**The following SMFM documents have been replaced effective 2024**:

Clinical Guideline #5: Twin‐twin transfusion syndrome

And

Consult Series #24: The importance of determining chorionicity in twin gestations

Replaced by Consult Series #72: Twin‐twin transfusion syndrome and twin anemia‐polycythemia sequence

**The following SMFM documents have been replaced effective 2025**:

Consult Series #36: Prenatal aneuploidy screening using cell‐free DNA

Replaced by Consult Series #74: Cell‐free DNA screening for aneuploidies: Updated guidance

**The following SMFM documents have been reaffirmed effective 2024**:

Consult Series #37: Diagnosis and management of vasa previa

Consult Series #41: The use of chromosomal microarray for prenatal diagnosis

Consult Series #42: The role of ultrasound in women who undergo cell‐free DNA screening

Consult Series #44: Management of bleeding in the late preterm period

Consult Series #45: Mild fetal ventriculomegaly: diagnosis, evaluation, and management

Consult Series #46: Evaluation and management of polyhydramnios

Consult Series #51: Thromboembolism prophylaxis for cesarean delivery

Consult Series #52: Diagnosis and management of fetal growth restriction

Consult Series #53: Intrahepatic cholestasis of pregnancy

Consult Series #54: Assessing the risk of maternal morbidity and mortality

Consult Series #55: Counseling women at increased risk of maternal morbidity and mortality

Consult Series #60: Management of pregnancies resulting from in vitro fertilization

Consult Series #61: Anticoagulation in pregnant patients with cardiac disease

Consult Series #62: Best practices in equitable care delivery‐ Addressing systemic racism and other social determinants of health as causes of obstetrical disparities

Consult Series #63: Cesarean scar ectopic pregnancy

Statement #8: Pharmacological treatment of gestational diabetes

Statement #9: Elective induction of labor in low‐risk nulliparous women at term: the ARRIVE trial

Statement #11: Antihypertensive therapy for mild chronic hypertension in pregnancy: The CHAP Trial

Fetal Anomalies Consult Series #3: Intracranial anomalies

Guideline #6: Fetal blood sampling

Guideline #9: Amniotic fluid embolism: diagnosis and management

**The following SMFM documents have been reaffirmed effective 2025**:

Consult Series #48: Immediate postpartum long‐acting reversible contraception for women at high risk for medical complications

Consult Series #50: The role of activity restriction in obstetric management

Consult Series #57: Evaluation and management of isolated soft ultrasound markers for aneuploidy in the second trimester

Consult Series #58: Use of antenatal corticosteroids for individuals at risk for late preterm delivery

Consult Series #59: The use of analgesia and anesthesia for maternal‐fetal procedures

Consult Series #64: Systemic lupus erythematosus in pregnancy

Consult Series #65: Transabdominal cerclage

Consult Series #66: Prepregnancy evaluation and pregnancy management of patients with solid organ transplants

Consult Series #67: Maternal sepsis

Statement: Response to the Food and Drug Administration's withdrawal of 17‐alpha hydroxyprogesterone caproate

Fetal Anomalies Consult Series #1: Facial anomalies

Guideline #8: The fetus at risk for anemia–diagnosis and management

Amniocentesis or chorionic villous sampling (CVS) checklist (online‐only)

Special Statement: Surgical safety checklists for cesarean delivery

Special Statement: A maternal transport briefing form and checklist

Special Statement: Operative vaginal delivery: Checklists for performance and documentation

Special Statement: Postpartum visit checklists for normal pregnancy and complicated pregnancy

Special Statement: Checklist for pregnancies resulting from in vitro fertilization

Special Statement: Checklists for transabdominal cerclage

Special Statement: Prophylactic low‐dose aspirin for preeclampsia prevention—quality metric and opportunities for quality improvement

Special Statement: Quality metrics for optimal timing of antenatal corticosteroid administration

Special Statement: Quality metric for timely postpartum follow‐up after severe hypertension

Special Statement: A quality metric for evaluating timely treatment of severe hypertension

Special Statement: Telemedicine in obstetrics—quality and safety considerations

Special Statement: Quality metric on the rate of postpartum diabetes screening after pregnancies with gestational diabetes mellitus

Special Statement: Checklist for thromboembolism prophylaxis after cesarean delivery

Special Statement: Checklist for postpartum discharge of women with hypertensive disorders

Special Statement: A critique of postpartum readmission rate as a quality metric

Special Statement: Cognitive bias and medical error in obstetrics—challenges and opportunities

Special Statement: Curriculum outline on patient safety and quality for maternal‐fetal medicine fellows

Special Statement: A critical examination of abortion terminology as it relates to access and quality of care

**All current SMFM clinical guidance can be accessed at** www.smfm.org/publications.

